# Long-term administration of ketamine induces erectile dysfunction by decreasing neuronal nitric oxide synthase on cavernous nerve and increasing corporal smooth muscle cell apoptosis in rats

**DOI:** 10.18632/oncotarget.10727

**Published:** 2016-07-20

**Authors:** Hung-Sheng Shang, Yi-No Wu, Chun-Hou Liao, Tzong-Shi Chiueh, Yuh-Feng Lin, Han-Sun Chiang

**Affiliations:** ^1^ Graduate Institute of Clinical of Medicine, College of Medicine, Taipei Medical University, Taipei, Taiwan; ^2^ Division of Clinical Pathology, Department of Pathology, Tri-Service General Hospital, National Defense Medical Center, Taipei, Taiwan; ^3^ Graduate Institute of Basic Medicine, Fu Jen Catholic University, New Taipei City, Taiwan; ^4^ Division of Urology, Department of Surgery, Cardinal Tien Hospital, Taipei City, Taiwan; ^5^ College of Medicine, Fu Jen Catholic University, Taipei City, Taiwan; ^6^ Department of Urology, Taipei Medical University Hospital, Taipei, Taiwan; ^7^ Division of Nephrology, Department of Medicine, Shuang Ho Hospital, School of Medicine, College of Medicine, Taipei Medical University, New Taipei City, Taiwan; ^8^ Division of Nephrology, Department of Medicine, Tri-Service General Hospital, National Defense Medical Center, Taipei, Taiwan

**Keywords:** apoptosis, corporal smooth muscle, erectile dysfunction, ketamine, nitric oxide synthase

## Abstract

We investigated and evaluated the mechanisms of erectile dysfunction (ED) in a rat model of long-term ketamine administration.

Adult male Sprague-Dawley rats (n = 32) were divided into four groups: namely the control group receiving intraperitoneal injection of saline, 1-month, 2-month and 3-month groups receiving daily intraperitoneal injection of ketamine (100 mg/kg/day) for 1, 2, and 3 month respectively. After treatment, animals underwent an erectile response protocol to assess intracavernosal pressure (ICP). Smooth muscle content was evaluated. Neuronal nitric oxide synthase (nNOS), inducible nitric oxide synthase (iNOS) and endothelial nitric oxide synthase (eNOS) expression were assessed using immunostaining assay. Ketamine-induced apoptosis was analyzed using TUNEL assay.

Long-term ketamine administration caused significantly decreased erectile responses as measured by ICP. Smooth muscle content was significantly decreased in the ketamine-treated rats for 3 months. In the erectile tissue, ketamine administration significantly reduced nNOS expression and increased iNOS content compared with controls, whereas eNOS expression was not altered. Ketamine induced apoptosis in corpus cavernosum.

The present study demonstrates that long-term ketamine administration led to erectile dysfunction in rat. The molecular mechanisms of ketamine-induced ED involved the increased apoptosis and up-regulated iNOS expression incorporating with loss of corporal smooth muscle content and reduced nNOS expression in cavernous nerve.

## INTRODUCTION

Erectile dysfunction (ED) is a common type of sexual dysfunction, which is characterized by an inability to achieve and maintain an erection for sexual performance. It is associated with aging and many common systemic disorders such as diabetes mellitus and hypertension [1, 2]. Aside from systemic diseases, a range of prescription drugs have been reported to contributed to ED in males including antihypertensive drugs, antidepressants and antiandrogens [3, 4]. In addition, recreational drug users have suffered from ED as a toxicological effect, including alcohol, heroin and ecstasy [5–7]. However, the relationship between long-term ketamine administration and ED remains unclear.

Ketamine is a N-Methyl D-Aspartate (NMDA) receptor antagonist which is commonly used to induce anesthesia, sedation, analgesia, and amnesia [8]. Abuse of ketamine has been reported to affect the urinary bladder, resulting in ulcerative cystitis [9]. Recent studies have stated that repeated application of ketamine in rats affects production of nitric oxide synthase (NOS) and induces apoptosis in the urothelial cell [10, 11]. Long-term use of ketamine has been shown to induce cardiotoxicity and neurodegeneration in vivo [12, 13]. Some studies have demonstrated that ketamine induces cytotoxicity in neurons through induction of apoptosis and changes of neuronal nitric oxide synthase (nNOS) expression [14, 15].

Nitric oxide (NO), a mediator of penile erection, is known to be synthesized by nNOS and endothelial nitric oxide synthase (eNOS) of cavernous nerve (CN) and endothelium in penis tissue. Impaired NO release in corpus cavernosum is associated with ED [16]. Inducible NOS (iNOS) has been demonstrated to be upregulated with age in rat penis in association with ED [17]. Although iNOS is not involved directly in physiological penile erection, it has been shown to play a role in defense mechanism against the aging or injury-associated fibrosis in the penile corpora cavernosa through impairing corporal smooth muscle to protect the erectile tissue by counteracting fibrosis [18].

In the present study, we investigated the impact of and explore the mechanisms of long-term ketamine administration on ED in a rat model by examining erectile response and histological and biochemical changes in CN and penile tissue.

## RESULTS

### Long-term administration of ketamine impaired erectile function in rats

**Figure 1:**
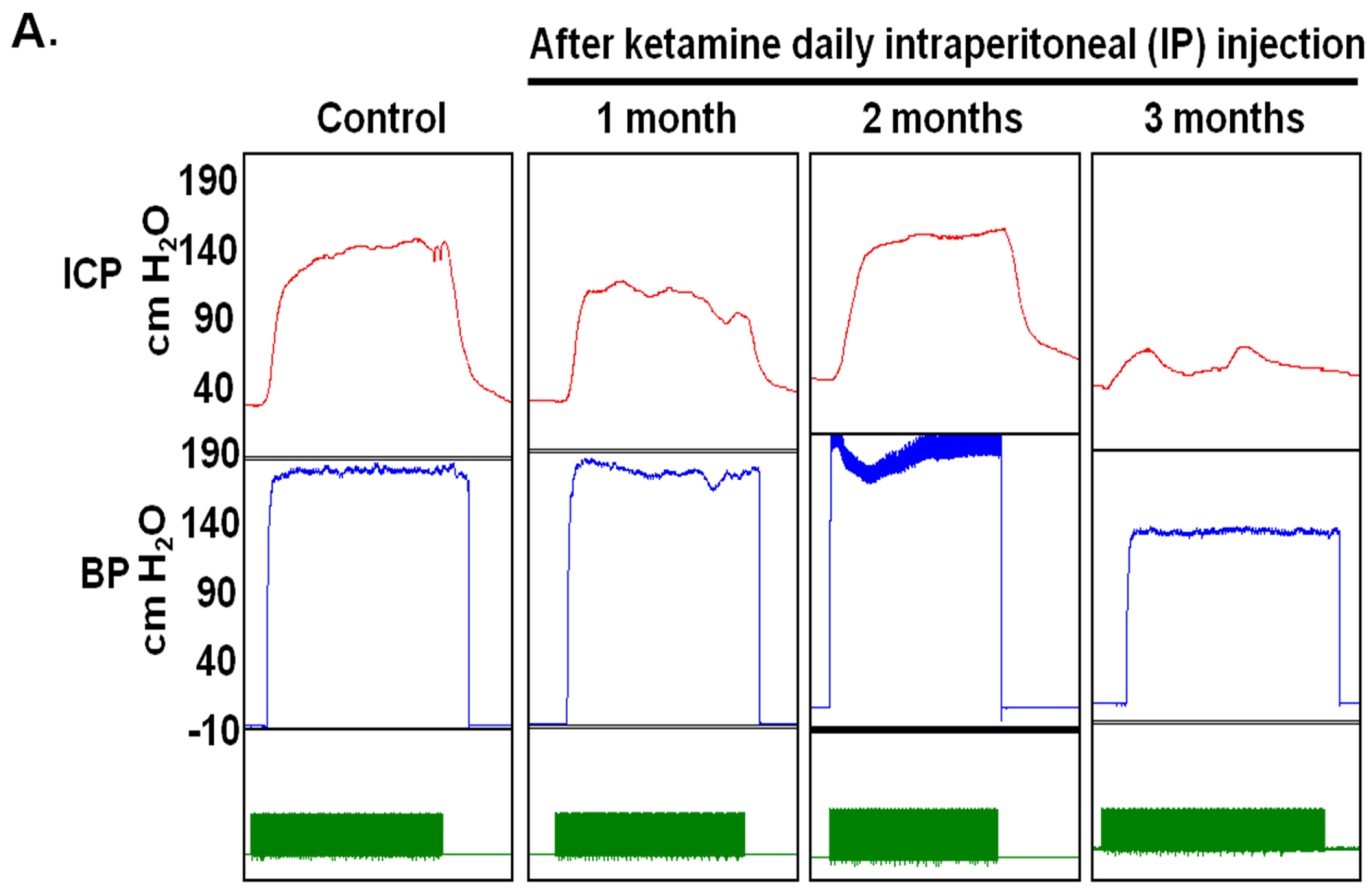

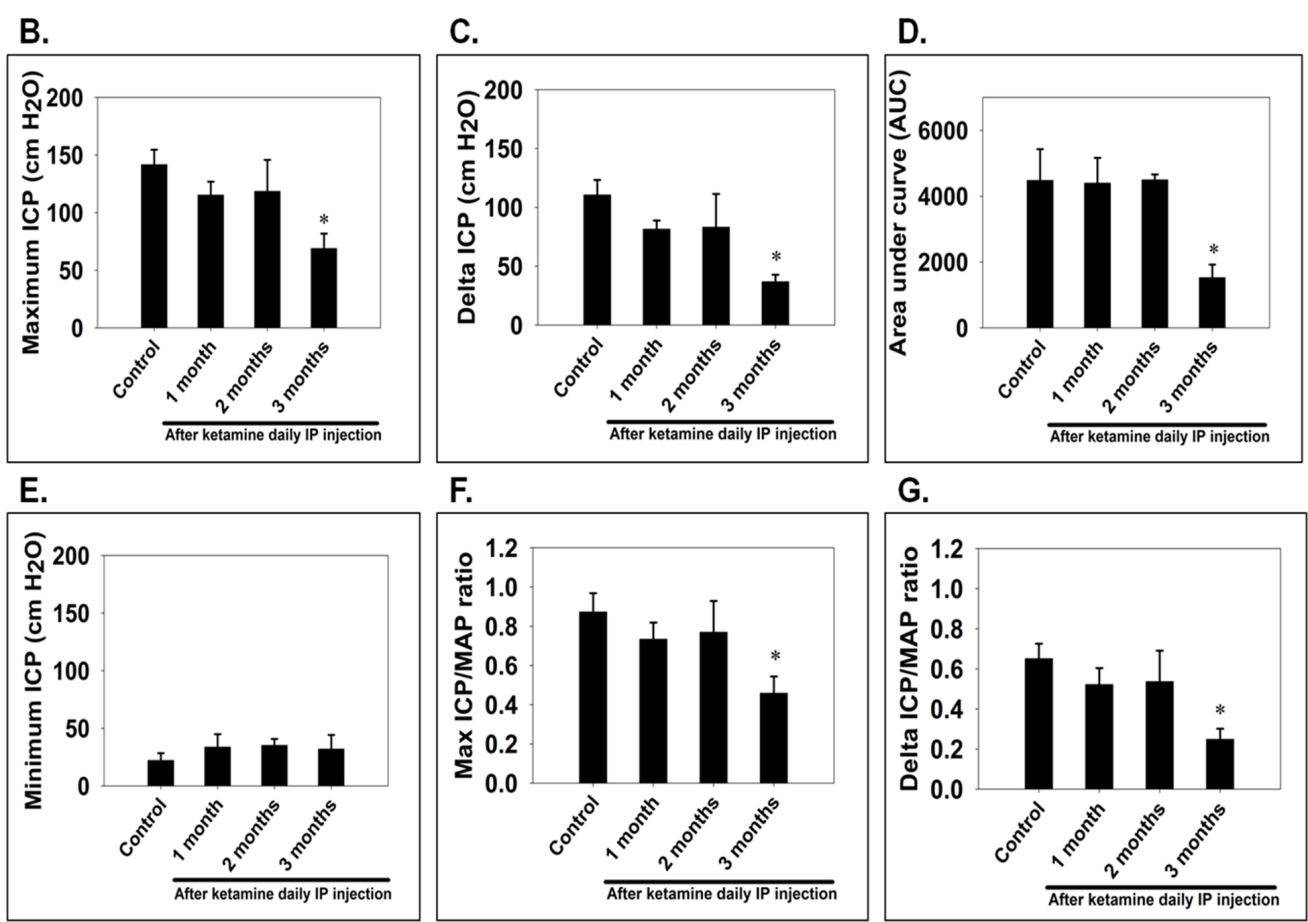
Electrostimulation of cavernous nerves after administration of ketamine for 1, 2 and 3 months **A.** Representative intracavernosal pressure (ICP) responses to 60 seconds for control or experimental groups stimulated at 1, 2 and 3 months after administration of ketamine. Mean maximal ICP **B.** delta ICP **C.** total ICP (area under the curve) **D.** and minimum ICP **E.** in each experimental group were measured. Ratio of maximal ICP to mean arterial pressure (MAP) **F.** and ratio of delta ICP to MAP **G.** were calculated for each group. Ketamine treated 3 months group showed significantly low ICP compared with control. Each bar depicts the mean ± SD for n = 8 animals per group. One-way ANOVA was used for statistical analysis.

To determine the effects of ketamine on erectile function, erectile response to ketamine was assessed by electro-stimulating the distal end of CN at 1, 2 and 3 months post-administration in all groups (Figure 1A). Ketamine-treated rats exhibited decreased maximum ICP, resulting in a significant reduction of ICP over a 3-month administration period (Figure 1B). As shown in Figure 1C, a significant change in ICP was observed in all ketamine-treated rats over a 3-month course of treatment. The rats receiving ketamine for 3 months displayed a significantly decreased level of total ICP (Figure 1D). Minimum ICP did not differ significantly among all experimental groups (Figure 1E). The ratio of maximum ICP or ΔICP to MAP were calculated and analyzed. There was a significant difference in maximum ICP/MAP ratio between the 3-month group and the controls (Figure 1F). The ratio of ΔICP to MAP was decreased in the rats treated with ketamine for 3 months (Figure 1G). All animal survived and no adverse clinical sign was observed.

### Long-term administration of ketamine decreased smooth muscle content of corpus cavernosum

**Figure 2:**
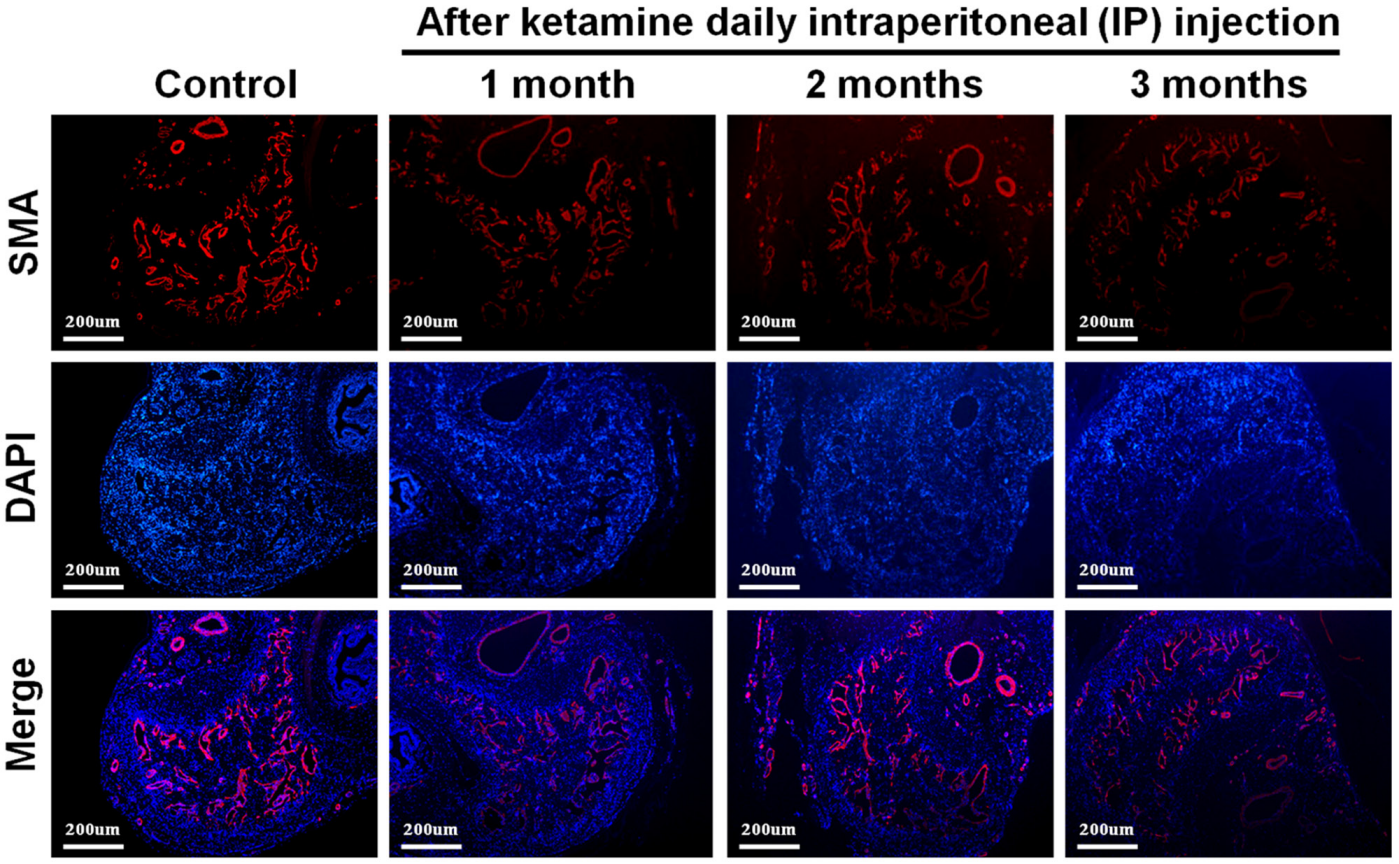
Representative fluorescent images of α-smooth muscle actin (SMA)-positive area in the rat penile corpus cavernosum (smooth muscle: red; nuclear: blue) Rats were treated without or with ketamine for 1, 2 and 3 month. The corpus cavernosum of penis was immunostained with α-SMA and 4′,6-diamidino-2-phenylindole. Ketamine treatment for 3 months showed the corporal smooth muscle atrophy compared with control. The original magnification was ×50.

**Figure 3:**
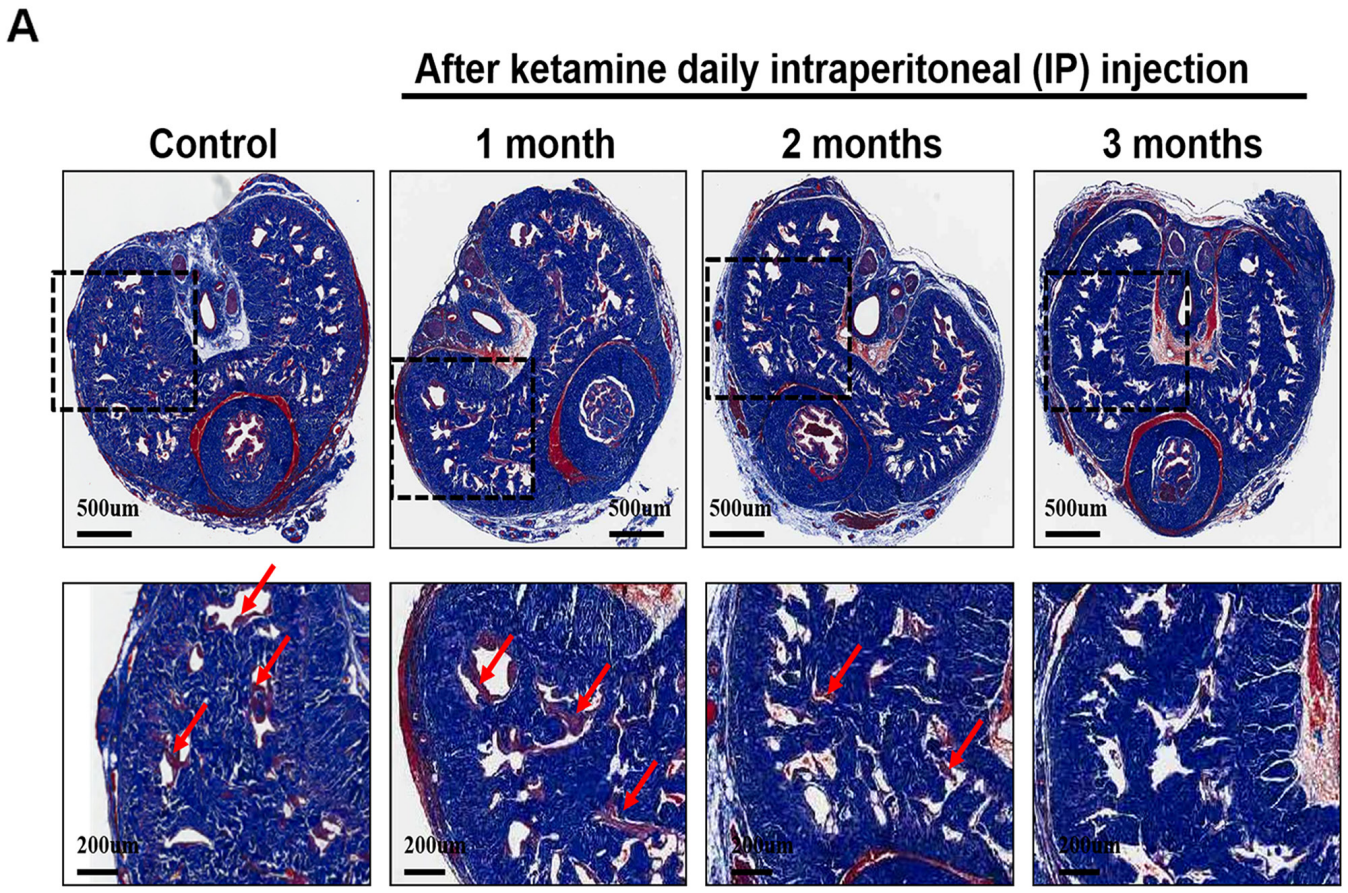

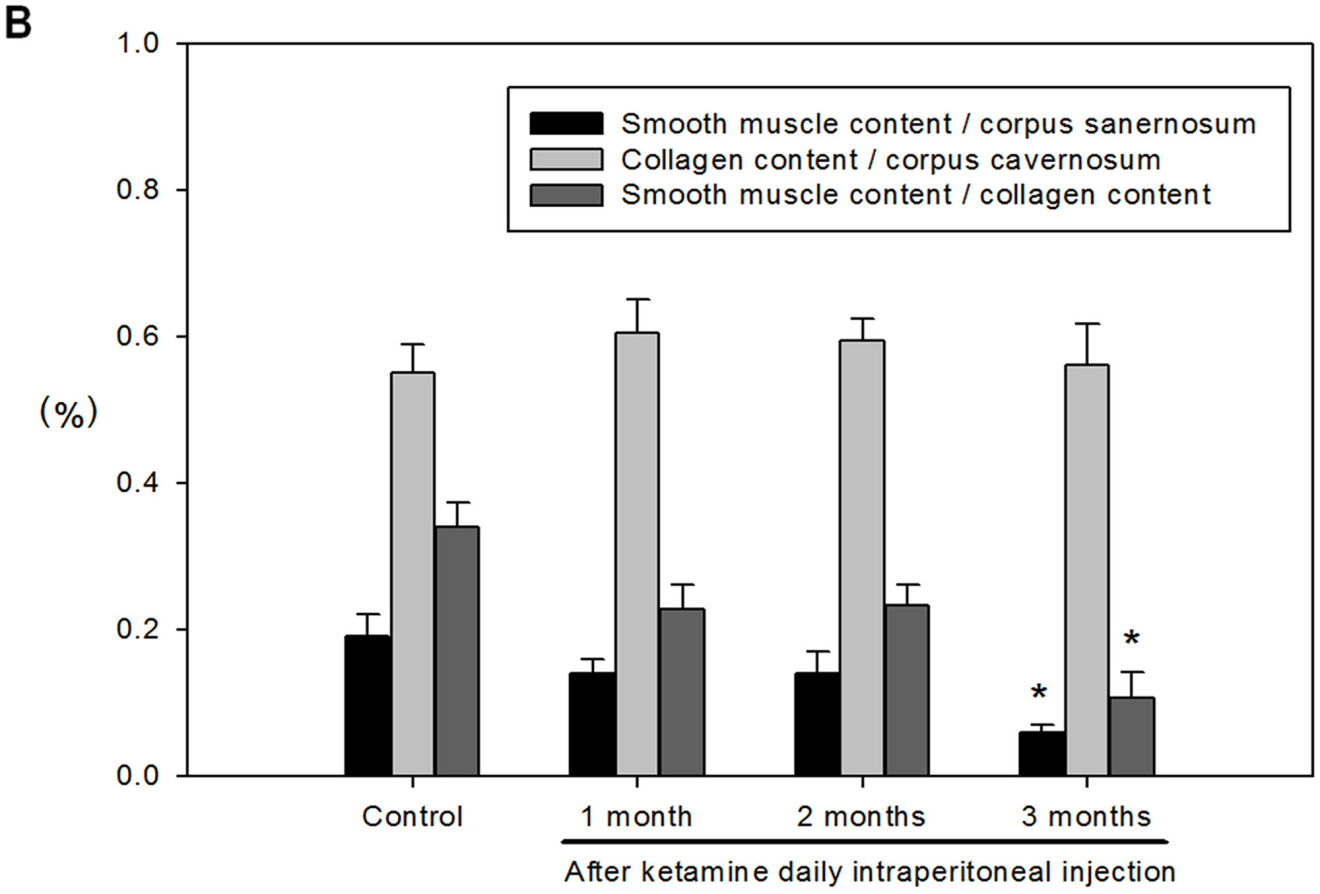
**A.** Representative images of the corpus cavernosum stained with Masson’s trichrome in all groups. Rats were treated without or with ketamine for 1, 2 and 3 month. The corpus cavernosum of penis was stained with Masson’s trichrome. In all images, the smooth muscle stains red, collagen and connective tissue stain blue and the cytoplasm stains a light pink. Ketamine treatment for 3 months, a significant decrease in smooth muscle content in corpus cavernosum was observed. Arrow is the corporal smooth muscle cell. **B.** The ratio of smooth muscle to collagen was as the index of fibrosis. Ketamine treatment for 3 months led to a significant increase in fibrosis compared to the controls. *p<0.05 response significantly different compared to control rats. The original magnifications were 20x and 100x, respectively.

We next investigated the influence of ketamine on smooth muscle content of corpus cavernosum. Immunofluorescence staining against α-SMA revealed that the area of corporal smooth muscle was decreased in response to ketamine administration in time-dependent manner (Figure 2 and Figure 3B). Masson’s trichrome staining for collagen and smooth muscle within the corpus cavernosum was performed. Following ketamine treatment for 3 months, a significant decrease in smooth muscle content in corpus cavernosum was observed (Figure 3A). The ratio of smooth muscle to collagen was as the index of fibrosis. Ketamine treatment for 3 months led to a significant increase in fibrosis compared to the controls (*p*<0.05). The area of corporal collagen content was unchanged with and without ketamine treatments (Figure 3B).

### Ketamine induced the decrease of nNOS–positive nerve fibers and stimulated the increase of iNOS expression in corpus cavernosum

**Figure 4:**
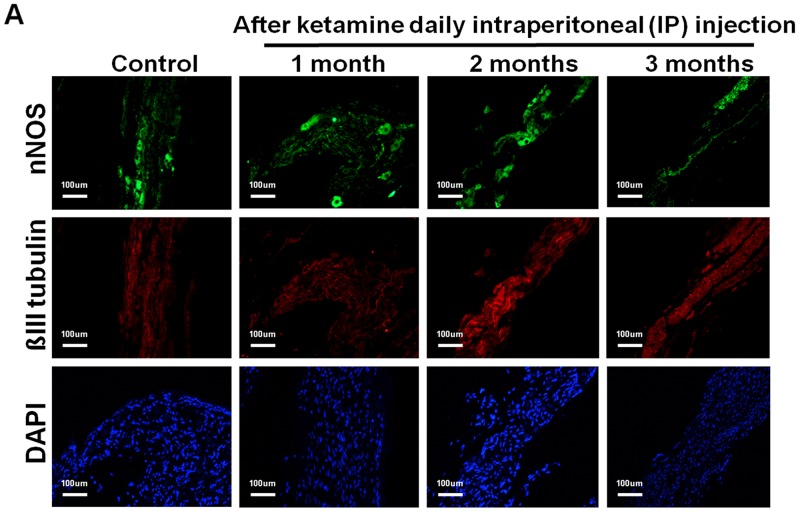

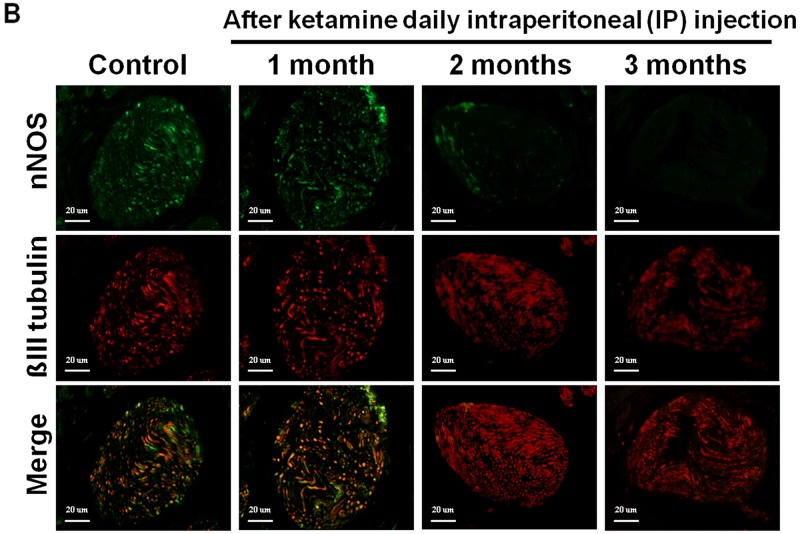

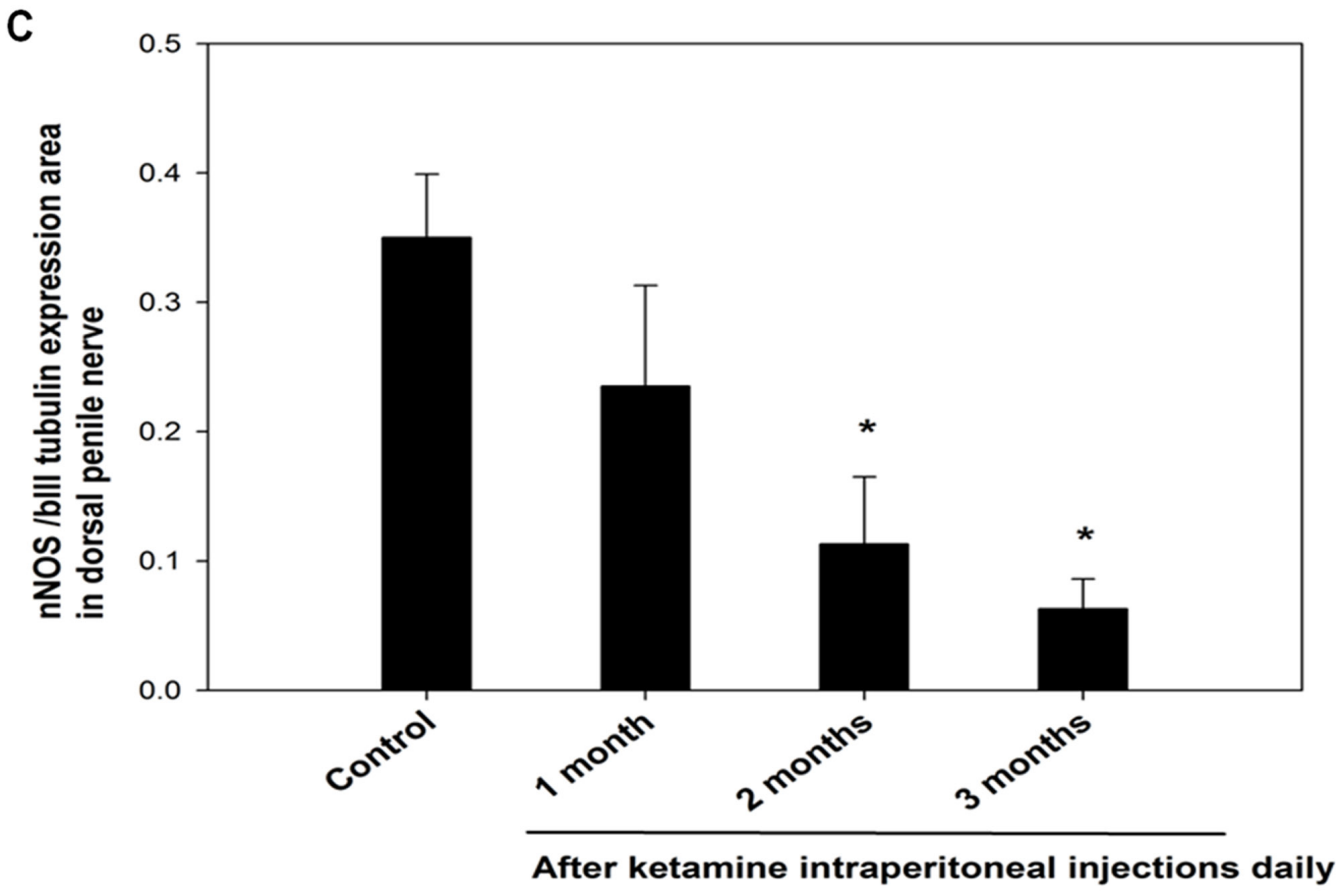
**A.** Immunofluorescence staining for neuronal nitric oxide synthase (nNOS) in the cavernous nerve (CN) of the control and ketamine treated groups, respectively. Representative images of the CN for each group are shown. (nNOS, green; β-III tubulin, red). The result showed that the number of nNOS-positive nerve fibers is dramatically reduced in the ketamine-treated 2 and 3 months groups compared with the control rats. Original magnification 100x. **B.** Immunofluorescence staining for nNOS in the dorsal penile nerve (DPN) of the control and ketamine treated groups, respectively. Representative images of the DPN from each group are shown. (nNOS, green; β-III tubulin, red). Original magnification 400×. **C.** Result of quantitative analysis. nNOS-positive nerve fibers in the DPN were quantified as the area of nNOS-positive nerve fibers/β-III-tubulin. The quantitative analysis showed that the number of nNOS-positive nerve fibers is decreased in ketamine-treated 2 and 3 months groups compared with the control group. *p < 0.05 versus control group.

**Figure 5:**
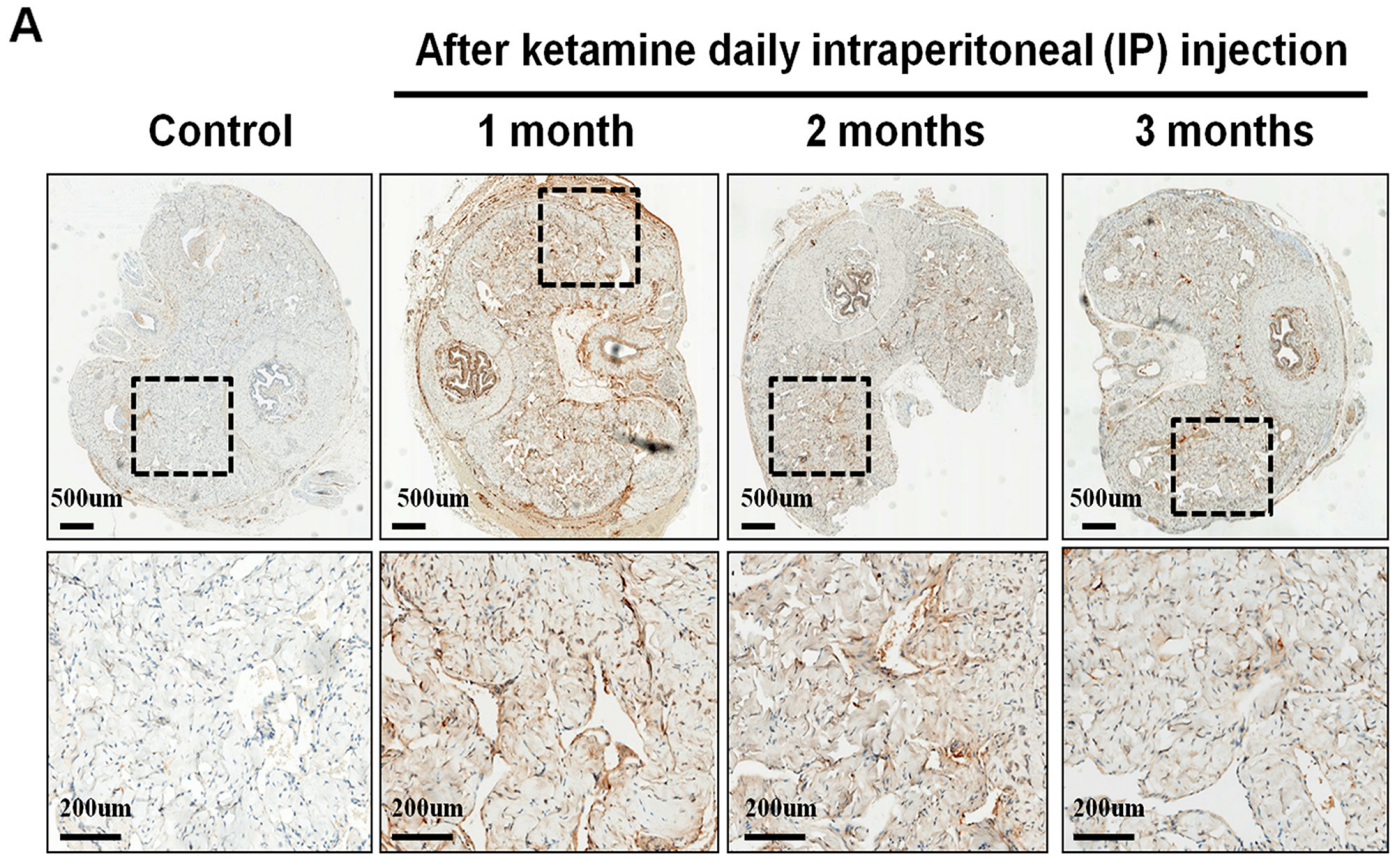

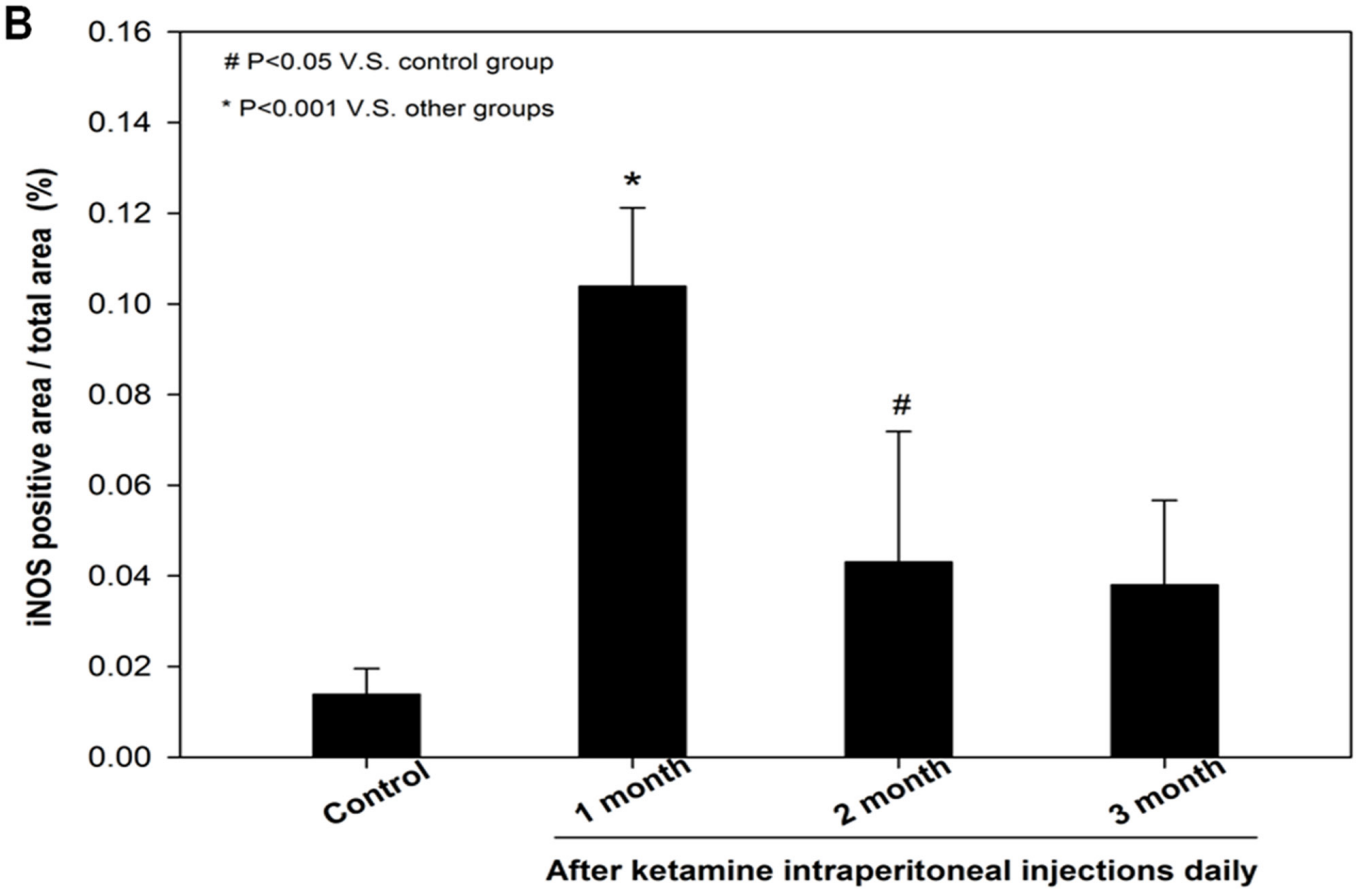
Immunohistochemistry staining for inducible NOS (iNOS) in corpus cavernosum Rats were treated without or with ketamine for 1, 2 and 3 months. The corpus cavernosum of penis was immunostained with iNOS. **A.** Representative images of the corpus cavernosum in each group. Original magnification ×400. **B.** Ratio of iNOS-positive cells within the corpora cavernosa in the four groups. There was a significant increase in the iNOS expression in ketamine treated 1 and 2 months groups (*p* < 0.001 and 0.05, respectively) compared with the control group. #*p* < 0.05 versus control group; *p < 0.001 versus other groups.

**Figure 6:**
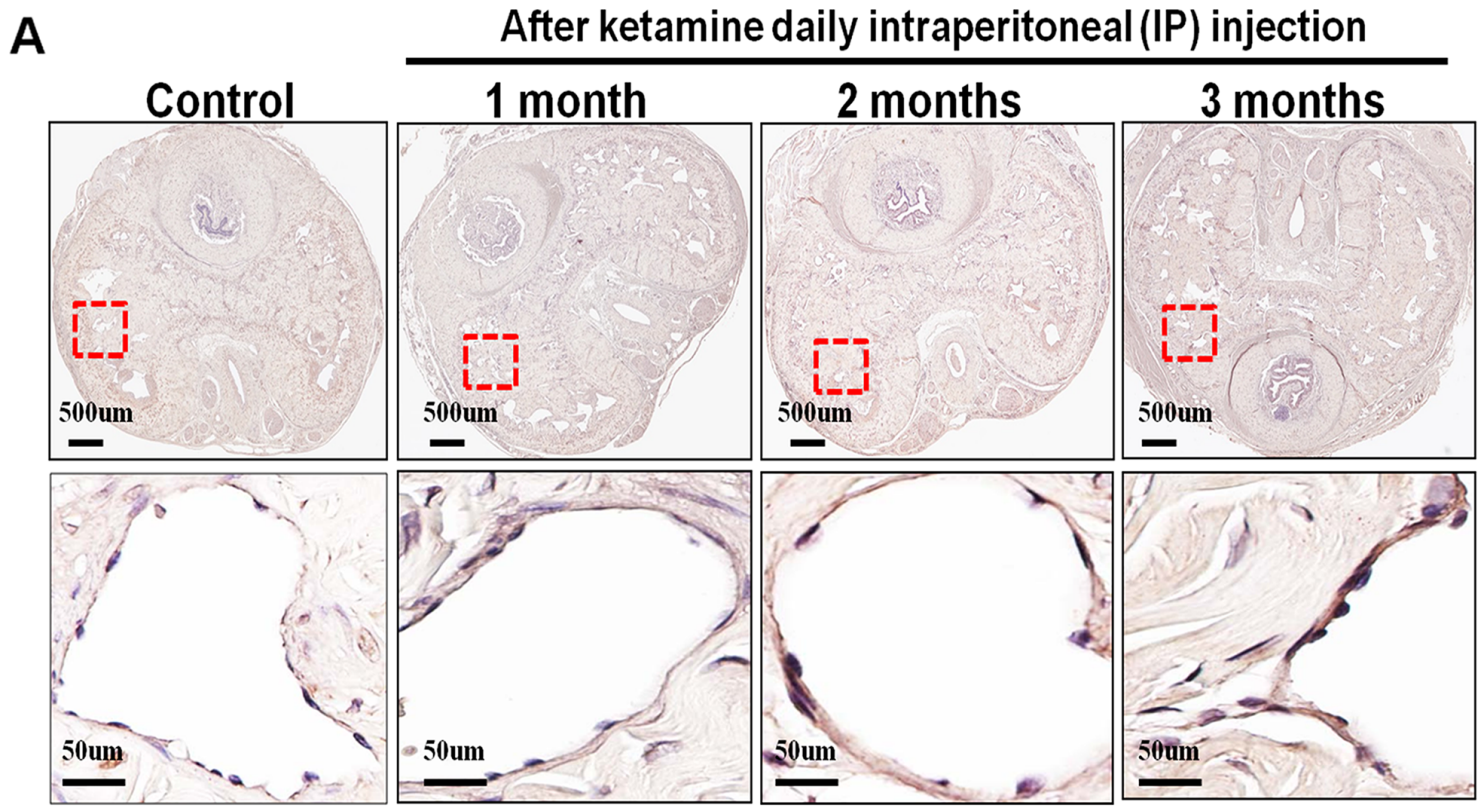

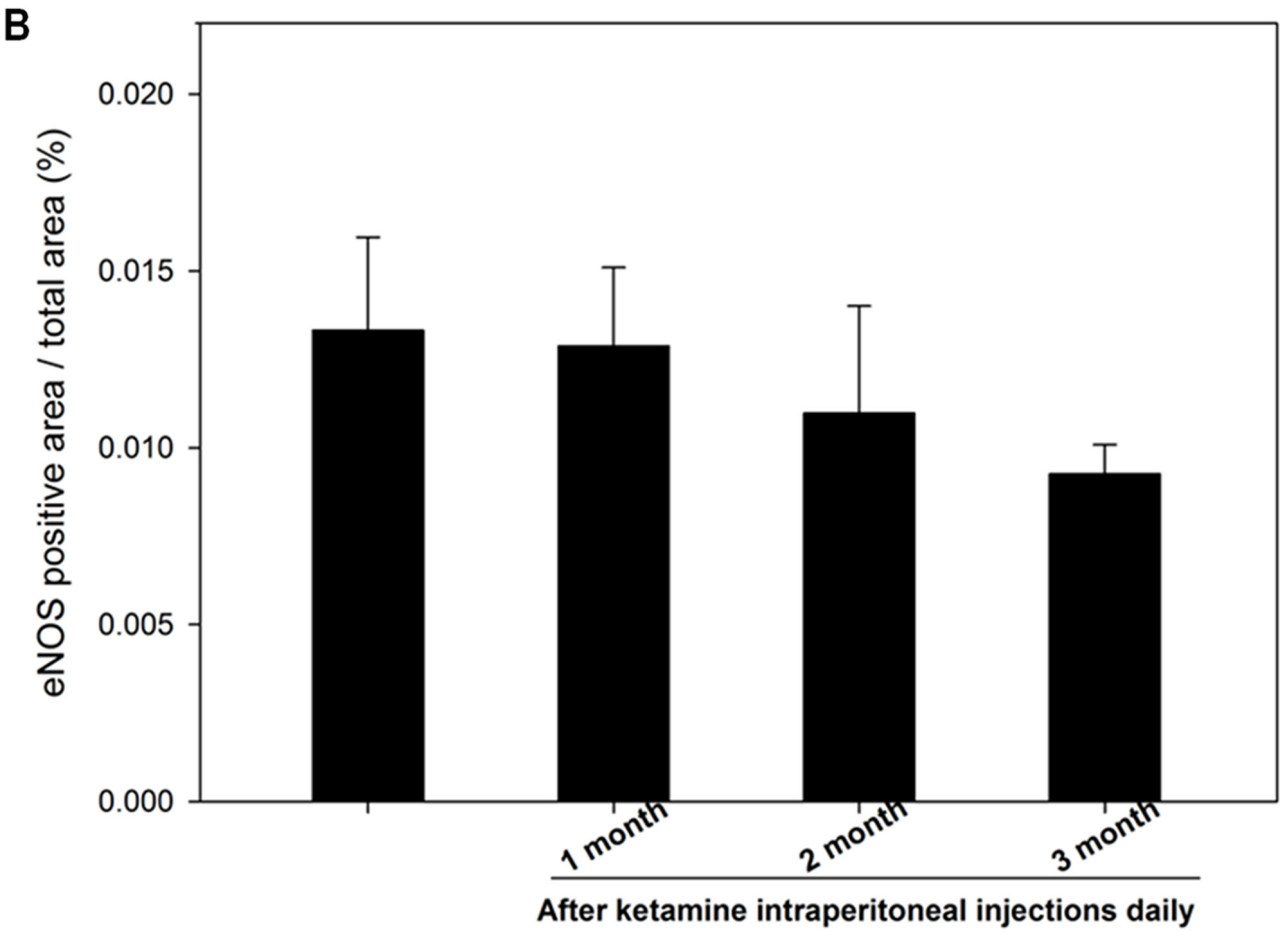
Immunohistochemistry staining for endothelial nitric oxide synthase (eNOS) in corpus cavernosum Rats were treated without or with ketamine for 1, 2 and 3 months. The corpus cavernosum of penis was immunostained with eNOS. **A.** Representative images of the corpus cavernosum in each group. Original magnification ×400. **B.** Ratio of eNOS-positive cells within the corpora cavernosa in the four groups. There was no difference between the four groups.

The nNOS-positive nerve fibers of the CN and dorsal penile nerve (DPN) were immunostained for β-III-tubulin to identify positive nerve fibers for nNOS and to quantify their nNOS content (representative images of each group in Figure 4A and 4B). Exposure to ketamine led to a reduction of nNOS expression in CN and DPN tissue. Using double-staining of nNOS together with βIII-tubulin, the ratio of the area of nNOS/β-III-tubulin expression was calculated. There was a significant decrease in nNOS expression in the rats treated for more than 2 months compared with that of controls (Figure 4C). In addition, iNOS expression in the corpus cavernosum without and with ketamine-treated groups was shown in Figure 5A. There was a significant increase in the iNOS expression in the 1 and 2 months ketamine treated group (*p* <0.001 and 0.05, respectively) compared with the control group but 3 months was not (Figure 5B). The changes in the expression of eNOS in corpus cavernosum were no significant among all treated groups over the period of study (Figure 6), although the expressions declined over time.

### Ketamine induces apoptosis in corpus cavernosum

**Figure 7:**
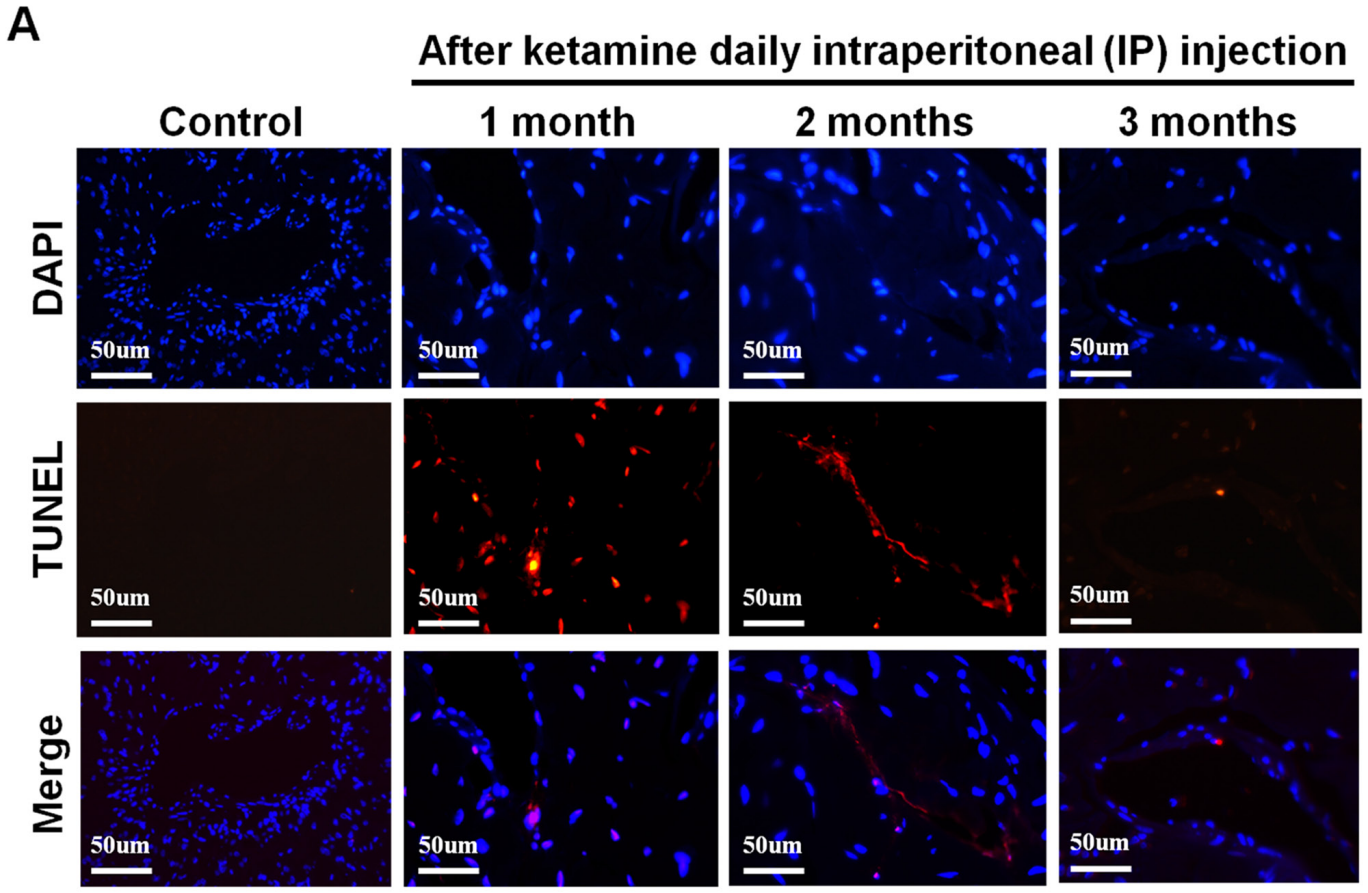

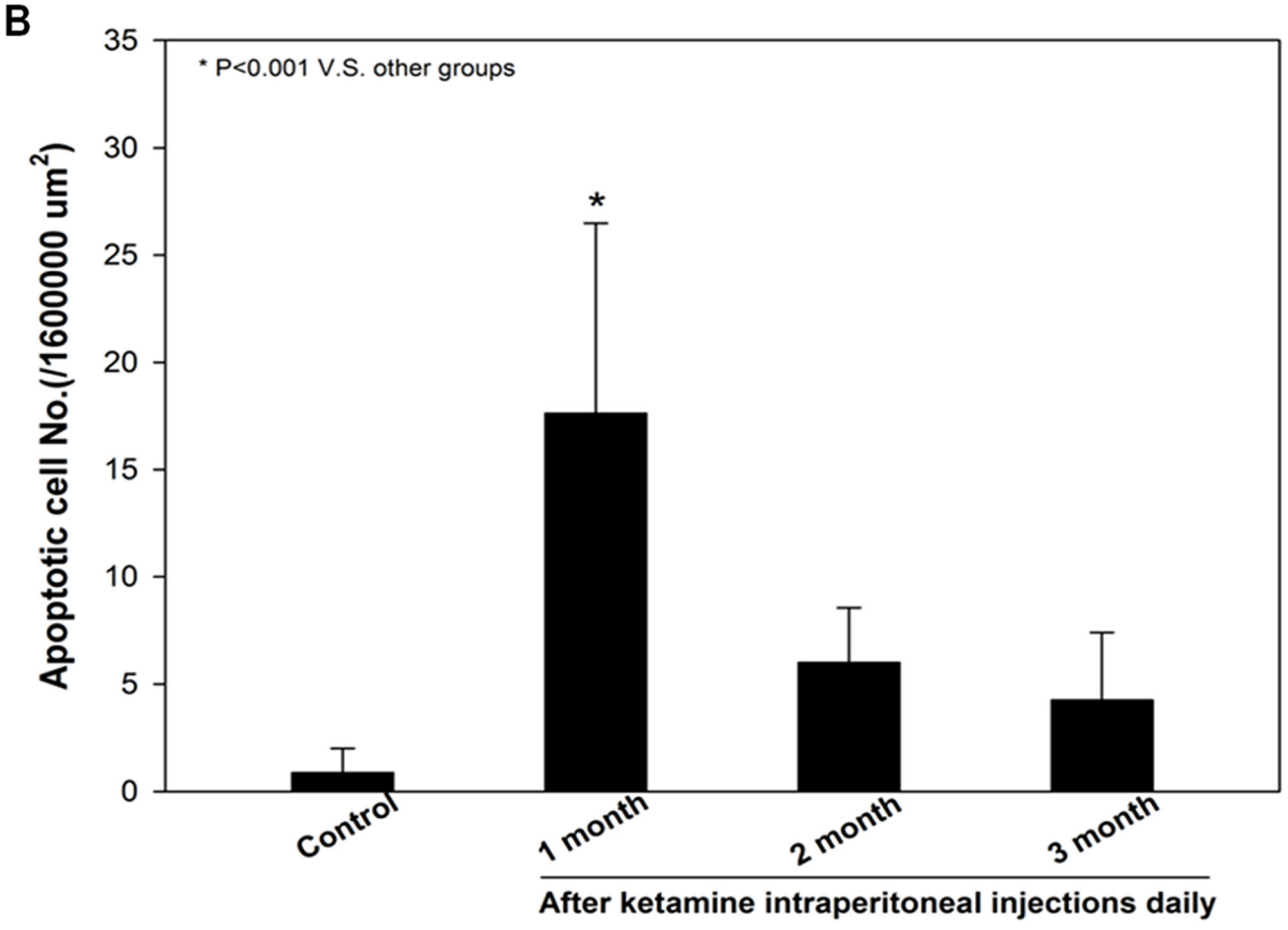

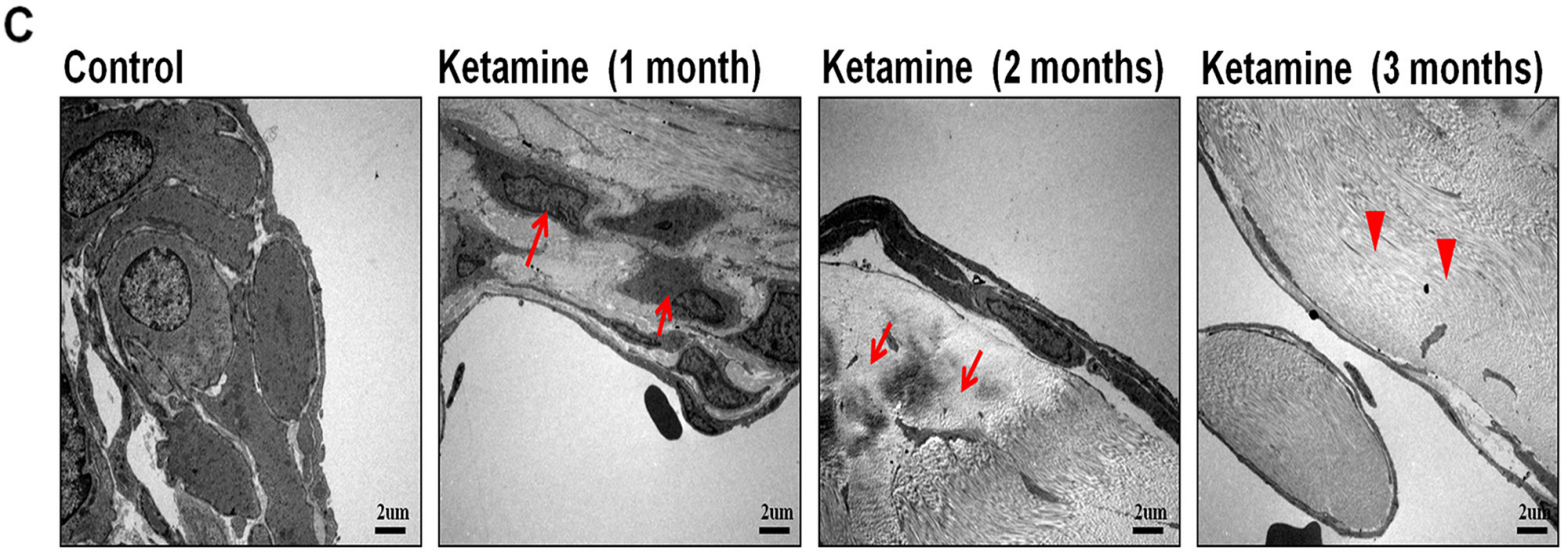
**A.** Transferase-mediated dUTP-biotin nick end labeling (TUNEL) staining from rats without or with ketamine treated for 1, 2 and 3 months showed nuclear colocalization with 4′, 6-diamidino-2-phenylindole (DAPI) (original magnification 200×). Only cells positive for both TUNEL and DAPI were considered positive for apoptosis. **B.** Ultrastructural analysis of the corpus cavernosum tissue from rats without or with ketamine treated for 1, 2 and 3 months. We observed abnormal structures and apoptosis of the smooth muscle cells at 1 month after ketamine treated and collagen deposition were clearly observed increase after ketamine treated 3 months. Arrow is corporal smooth muscle site; Arrow head is the collagen deposition (original magnification 5000×). **C.** Apoptotic cell quantification expressed as the number of apoptotic cell/area of the corpus cavernosum. Apoptotic index in ketamine treated 1 month significantly increase increased compared with that in others groups. *p < 0.001 compared with other groups.

As decreased smooth muscle content, we examined the mechanism underlying the ketamine-induced toxicity using TUNEL assay. Apoptotic cells in corpus cavernosum were significantly more abundant in animals that treated ketamine 1 month compared with control animals. However, prolonged exposure to ketamine for 2 and 3 months resulted in no significant increase in apoptotic cell number (Figure 7A and 7C). TEM revealed ultrastructural features of the corpus cavernosum and relationships between the smooth muscle cells (SMCs) and the endothelium adjacent to the SMCs during the course of ketamine treatment. TEM images revealed healthy smooth muscle of the corpus cavernosum near the sinusoid and along the boundary of the endothelial cells and close to the collagen tissue in the control groups. However, abnormal structures and apoptosis of the SMCs were observed in the 1-month ketamine-treated group. After 3-month ketamine treatment, a complex extracellular matrix like collagen was observed for an increase near endothelial cells, whereas endothelium was not affected in all treated groups (Figure 7B).

## DISCUSSION

In this study, we investigated the toxic effects of ketamine in reproduction system with focus on erectile tissues. We showed that long-term ketamine administration deteriorated erectile function. Ketamine treatment resulted in decreased nNOS positive nerve fibers and loss of smooth muscle content in erectile tissue. Moreover, ketamine increased iNOS production in corpus cavernosum, which was accompanied with elevated degree of apoptosis at early stage after ketamine treatment.

Recreational use of ketamine implying repeated administration had been reported to be associated with adverse consequences including lower urinary tract symptoms, impaired consciousness, abdominal pain, and dizziness [9, 19–20]. Preclinical studies also stated that long-term administration of ketamine induces toxicity in central nervous system, cardiovascular system and urinary system [21–23]. In current study, we reported that repeated administration of ketamine affected erectile physiology in rats. Our data supported the clinical findings that use of ketamine leads to incompatibility with sex [24, 25].

An erection requires a sequence of events involving a precise coordination of functioning nerves, arteries and veins. A concatenation of conditions and events has been shown to contribute to impotence [26, 27]. As corporal smooth muscle controls the vascular event leading to erection, change of smooth muscle content and ultrastructure is associated with affected erectile response [28–30]. Previous study had reported a significant difference in the percentage of corporal smooth muscle between normal and impotent subjects [31]. Our data showed that long-term administration of ketamine induced a significant loss of smooth muscle content corresponding to the degree of ED. It is suggested that ketamine exerts toxic effects on corporal smooth muscle leading to ED.

Nitric oxide, a physiologic mediator of erectile function is catalyzed by NOS localized to rat penile neurons innervating the corpora cavernosa and neuronal plexuses in the adventitial layer of penile arteries [32]. Among three isoforms of nNOS and eNOS are in principal involved in the initiation of penile erection [33]. nNOS is found in the CNs, the DPN and the adventitia of penile arteries and has been reported to be the major mediators of penile erection compare to eNOS [34, 35]. It has been suggested that nNOS initiates the erectile response, which is subsequently maintained and augmented by eNOS activity [32, 36]. Our results showed that expression of nNOS was decreased in ketamine treated rats in a time-dependent manner corresponding to degrees of ED. The findings are consistent with previous studies, suggesting that ketamine-induced ED is attributed to the impairment of nNOS expression as a result of nerve damages [25, 37]. Interestingly, immunohistochemistry assay revealed that there was no significant change in eNOS content. Role of eNOS in erectile process has been shown to maintain erectile response through release of endothelial NO for vasodilation [38, 39]. As the level of eNOS was not altered, the effects of ketamine on eNOS activity in corpus cavernosum are questionable and require further studies to elucidate.

Despite increasing evidence showing the role of nNOS and eNOS in erection, iNOS has not been examined for its role in physiological erection as well as related mechanism [40–41]. iNOS is shown to modulate critical features of inflammation, neovascularization, and collagen deposition on the fibrovascular tissue induced by sponge implants in mice [42]. It has been reported that use of selective iNOS inhibitor in rats with the Peyronie’s disease-like plaque led to fibrosis with collagen deposition [43]. Our results revealed that ketamine treatment for 1 month increased the expression of iNOS in the corpus cavernosum, suggesting that ketamine induces inflammation and attempted to anti-fibrosis in erectile tissue at early stage of ketamine administration. However, collagen deposition on the corpus cavernosum tissue was accompanied with a decreased iNOS expression.

Apoptosis is a programmed biological event to remove unwanted cells in physiological and pathological conditions. Previous studies have reported that denervation of the rat penis induced apoptosis of penile erectile tissue in diabetes and nerve injury rats [44, 45]. Inhibition of apoptosis in the corpus cavernosum has been demonstrated to improve ED [46, 47]. Increasing evidence had highlighted the involvement of apoptosis in ketamine-induced toxicity in various types of cell, including cardiac endothelial, renal epithelial, neural and bladder epithelial cells [12, 48–50]. In this study, we showed that ketamine treatment for 1 month induced apoptosis in the corpus cavernosum. The pro-apoptotic effect of NO produced by iNOS has been also reported both in spinal cord ischemia/reperfusion injury rat and in the microvasculature [51, 52]. It is agreed that increase of iNOS expression involved in apoptosis pathological conditions [53, 54]. Given these findings, it is postulated that ketamine induces apoptosis in corporal cavernosum through up-regulating iNOS expression as results of excessive NO release. Because of corporal smooth muscle atrophy, prolonged ketamine treatment showed that apoptotic cell numbers are time-dependently decreased in our study. The TEM result was in accord with TUNEL and iNOS, ketamine injection induced a large number loss of cells at the early stage and collagen deposition with fibrosis after a long-term ketamine treatment.

In conclusion, the present study demonstrates that long-term ketamine administration has adverse effects on erectile function in rat. The molecular mechanisms of ketamine-induced ED involved the activation of apoptosis by up-regulating of iNOS cause the loss of corporal smooth muscle content and reduce of nNOS expression on CN. Further studies are required to determine the apoptotic pathways, which are influenced by ketamine in chronic setting.

## MATERIALS AND METHODS

### Animals

Thirty-two 8-week-old, male, Sprague Dawley rats (weight, 250-300g) were used in this study. All animals were obtained from BioLasco Taiwan Co., Ltd. (Taipei, Taiwan), and the study protocol was reviewed and approved by the National Defense Medical Center Animal Care and Use Committee (IACUC approval NO: IACUC-14-168)

### Ketamine administration procedures

Rats were maintained in a standard cage environment at 25°C and exposed to a 12 hour light and dark cycle. Animals were randomly divided into four groups, namely the control group receiving intraperitoneal injection of saline, 1-month, 2-month and 3-month groups receiving daily intraperitoneal injection of ketamine (100 mg/kg/day) (Imalgene 1000®, Merial, Lyon, France), for 1, 2, and 3 month respectively.

### Measurement of erectile responses

After treatment, the CNs were exposed and isolated via a repeat midline abdominal incision, and the crura of the penis were identified. A 24G needle containing 50 U/mL of heparin solution was inserted into the right penile crus and connected to a polyethylene-50 tubing to measure intracavernosal pressure (ICP) with an MP36 pressure transducer (Biopac Systems Inc., Santa Barbara, CA, USA) and Biopac Student Lab (BSL) 3.7.3 software (Biopac Systems Inc., Santa Barbara, CA, USA). The CNs were stimulated using a bipolar, stainless steel electrode through monophasic, rectangular pulses generated by a computer with a DS3 constant–current-isolated stimulator (AutoMate Scientific Inc., Berkeley, CA, USA). The stimulus parameters included 7.5-mA amplitude, 20-Hz frequency, 0.2-ms pulse width, and 60-s duration. A real-time response of the erectile tissue was determined based on the maximum ICP, the change in ICP (ΔICP), the area under the ICP curve (AUC), and the ratio of change in ICP and the maximum ICP and mean arterial pressure (MAP; ΔICP/MAP; maximum ICP/MAP). The data were obtained from 5 replicates continuously on erectile responses measurement in each rat.

### Immunofluorescence staining

After measuring the erectile response, the animals were sacrificed by administering a high dose of pentobarbital sodium solution. The CNs and tissue from the middle portion of the penis of rats were obtained. The freshly dissected tissue was formalin-fixed (10% formaldehyde w/v), dehydrated, postfixed, and embedded. 5-μm-thick cross-sections were prepared followed by 10 minutes deparaffinization in xylene for twice and hydration of sections through graded alcohols. The slides were blocked with 10% goat serum/2% bovine serum albumin (BSA)/0.2% Triton X-100 (Sigma-Aldrich, St. Louis, MO, USA) for 20 minutes. Resulting slides were incubated overnight at room temperature with rabbit anti- nNOS (Santa Cruz Biotechnology, Santa Cruz, CA, USA), mouse anti-neuron-specific β-III tubulin, and anti- α-smooth muscle actin (α-SMA) antibodies (both from Abcam Inc., Cambridge, MA, USA), followed by a 3-hour incubation in 1:400 dilution of the secondary antibody, conjugated with AlexaFluor 488 or Texas Red (Invitrogen, Carlsbad, CA, USA). The slides were evaluated using fluorescence microscopy. For analysis of nNOS content, the ratio of the area of nNOS-positive fibers per nerve over the β-III-tubulin area of the nerve at a magnification of ×400 was calculated. Staining was repeated triplicate in each tissue sample

### Histochemistry and immunohistochemistry staining

Penile tissue sections were prepared as aforementioned and adjacent tissue sections were used for: (a) Masson trichrome staining for collagen (blue) and smooth muscle cell (red) (Muto Pure Chemicals, Tokyo, Japan); (b) immunodetection with: (i) polyclonal antibody against iNOS (Calbiochem, La Jolla, CA, USA); (ii) rabbit polyclonal anti-eNOS IgG antibody against eNOS (Santa Cruz Biotechnology, Santa Cruz, California, USA). All the staining procedure were carried out according to the manufacturer’s instructions.

### Transferase-Mediated dUTP-biotin Nick End Labeling (TUNEL) Staining

Quantification of apoptotic cells was performed by detecting DNA damage in-situ using Apo-BrdU In Situ DNA Fragmentation Assay Kit (Biovision Inc., Milpitas, CA, USA) and counterstaining by 4′,6-diamidino-2-phenylindole (DAPI) in paraffin-embedded tissue sections. The slides were evaluated using fluorescence microscopy.Quantitative analysis for apoptotic cell and all computerized histomorphometric analyses of the nerve were performed using IMAGEJ (National Institutes of Health, Bethesda, Maryland, USA, http://imagej.nih.gov/ij/) software 1.46r by cell biologists.

### Transmission electron microscopy (TEM) assay

Animals were sacrificed by administration of a high dose of pentobarbital sodium solution. Tissue from the middle portion of the penis, were obtained from each rats. All tissue samples were cut into small pieces, fixed in 2.5% phosphate-buffered glutaraldehyde (0.1 M, pH 7.2) overnight, and post-fixed in 1% phosphate-buffered osmium tetroxide (0.1 M, pH 7.2). The specimen were dehydrated through a graded concentration of ethanol, and embedded in Epon-812 (Sigma-Aldrich, St. Louis, MO, USA). One-micron semi-thin sections were stained with toluidine blue. Ultra-thin sections from selected blocks were stained with uranyl acetate and lead citrate, and observed under a JEOL JEM-1400 transmission electron microscope (Jeol Ltd., Tokyo, Japan).

### Statistical analysis

Data were expressed as mean standard deviation. Treatment groups were compared using one-way analysis of variance and the Scheffe post hoc test with statistical significance indicated at *p* < 0.05. The software used for statistical analysis was SPSS version 12.0 (SPSS Inc., Chicago, IL, USA) for Windows.
